# Polarization-selective four-wave mixing in a degenerate multi-level system

**DOI:** 10.1038/s41598-024-56229-5

**Published:** 2024-03-08

**Authors:** Jaeuk Baek, Sanghyun Park, Min-Hwan Lee, Heung-Ryoul Noh, Geol Moon

**Affiliations:** 1 Department of Physics, Chonnam National University, Gwangju, 61186 Korea; 2 Center for Quantum Technologies, Chonnam National University, Gwangju, 61186 Korea

**Keywords:** Atomic and molecular interactions with photons, Nonlinear optics, Quantum optics, Atomic and molecular interactions with photons, Nonlinear optics, Quantum optics

## Abstract

This paper describes the first observation of polarization-selective four-wave mixing signals in conventional coupling-probe spectroscopy, specifically, saturation absorption spectroscopy in ^85^Rb atoms. The four-wave mixing signal is induced by two counter-propagating laser beams in a degenerate multi-level atomic system, involving the $F_g=3 \rightarrow F_e =2,3$, and 4 transitions of the ^85^Rb D2 line. Consequently, the four-wave mixing signals copropagating along the probe beam induce polarization rotation of a linearly polarized probe beam. To distinguish these four-wave mixing signals from the resulting probe beam, we detect the polarization components orthogonal to the polarization direction of the input probe beam, depending on the linear polarization angles between the probe and coupling beams. The experimental findings demonstrate excellent agreement with theoretical results.

## Introduction

The four-wave mixing (FWM) phenomenon has been widely explored owing to its potential applications in quantum computation^1^, quantum communication^2^, quantum metrology^3–10^, and fundamental quantum physics testing^11,12^ in degenerate two-level atomic energy structures^13–27^ and nondegenerate multi-level atomic energy structures^28–41^. These experiments have been performed under various conditions, such as the number of optical fields, optical frequencies, optical polarizations, propagation directions of optical fields, atomic energy level configurations, and external magnetic perturbations.

The interactions of pure or degenerate two-level atomic systems with two laser fields represent a well-known problem in atomic and laser spectroscopy. Typical examples of this scenario include Doppler-free spectroscopy experiments, such as saturated absorption spectroscopy (SAS)^42–44^ and polarization spectroscopy (PS)^45,46^. When a two-level atom simultaneously interacts with two laser beams, i.e., a coupling beam with frequency $\omega _c$ and a probe beam with frequency $\omega _p$, the matrix elements of the density operator $\rho$ between the excited ($\left| e\right\rangle$) and ground ($\left| g\right\rangle$) states can be expressed as follows^47^:

1 $$\begin{aligned} \rho _{eg} = \rho _{eg}^{(1)} e^{-i \omega _p t} +\rho _{eg}^{(2)} e^{-i \omega _c t} +\rho _{eg}^{(3)} e^{-i \left( 2 \omega _c -\omega _p \right) t} +\rho _{eg}^{(4)} e^{-i \left( 2 \omega _p -\omega _c \right) t}+\cdots , \end{aligned}$$

where $\rho _{eg} \equiv \left\langle e \right| \rho \left| g \right\rangle$. In Eq. (1), the component $\rho _{eg}^{(1)}$ ($\rho _{eg}^{(2)}$) corresponds to the absorption and dispersion of the probe (coupling) beam. The components $\rho _{eg}^{(3)}$ and $\rho _{eg}^{(4)}$ describe the two FWM signals. The wave vectors of the FWM signals associated with $\rho _{eg}^{(3)}$ and $\rho _{eg}^{(4)}$ are $2 {\vec k}_c -{\vec k}_p$ and $2 {\vec k}_p -{\vec k}_c$, respectively, where ${\vec k}_p$ (${\vec k}_c$) is the wave vector of the probe (coupling) beam. Observation of these two FWM signals in the co-propagating geometry were reported^14,22^.

The effect of $\rho _{eg}^{(1)}$ in a pure two-level atomic system can be detected by measuring the absorption of the probe beam. However, in the case of a degenerate two-level atomic system, the nonlinear characteristics can also be detected by measuring the rotation of the probe beam with respect to the propagation direction. To induce this rotation in the polarization of the probe beam, a new field with its electric field perpendicular to that of the probe beam is required. As discussed in theoretical description, this new field, with a polarization axis perpendicular to that of the probe beam, is generated through nonlinear interactions of photons, involving at least two coupling photons and one probe photon interacting with atoms. Thus, this nonlinear optical process can be interpreted in terms of the FWM, as a new field is generated from interactions among three photons via atoms. Because this field can be detected only along the axis perpendicular to that of the probe beam, it is termed the polarization-selective FWM signal.

This study focuses on polarization-selective FWM signals in completely overlapped counter-propagating coupling-probe spectroscopy, such as SAS in ^85^Rb atoms. We selectively extract the pure FWM signals copropagating with the probe beam using a polarizer. Notably, this experimental observation cannot be understood using a simple two-level model. The atoms of interest are subjected to a cycling transition involving degenerate Zeeman sublevels in a complicated optical polarization configuration. Although we report the observation of FWM signals in the counter-propagating geometry, they can also be observed using the copropagating geometry, given that the energy and momentum are conserved in such a configuration. It is essential to emphasize that the FWM signals associated with $\rho _{eg}^{(3)}$ and $\rho _{eg}^{(4)}$ are observed only in the copropagating geometry^14,22^.

## Theory

### Mechanism for FWM generation

**Figure 1:**
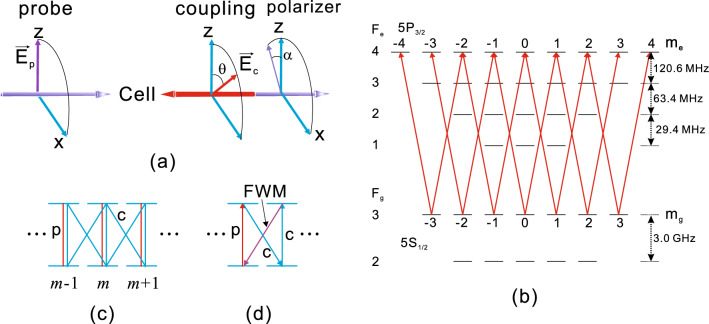
(**a**) Simplified schematic of coupling-probe spectroscopy. The laser beams are linearly polarized, and the probe beam passing through a polarizer with a polarization angle $\alpha$ is detected. (**b**) Energy level diagram for the ^85^Rb D2 line. The red arrows represent excitation by the laser beams where the quantization axis is chosen as the propagation direction of the lasers. (**c**) Part of an energy level diagram with Zeeman magnetic sublevels. The excitations by the coupling and probe beams are denoted as c and p, respectively. (**d**) Simple diagram for the FWM signal generation.

This section describes the generation mechanism of FWM signals in the coupling-probe spectroscopy geometry applied to ^85^Rb atomic vapor. A simplified schematic diagram is presented in Fig. 1a. The probe and coupling beams propagate along the *y* axis in opposite directions and overlap in an atomic cell. The polarization vectors for the probe and coupling beams are defined as $\hat{z}$ and $\hat{z} \cos \theta + \hat{x} \sin \theta$, respectively. After traversing a cell, the polarization vector of the probe beam is rotated slightly with respect to the *z* axis due to the anisotropy induced in the atomic cell. Subsequently, the probe beam passes through a polarizer with a transmission angle $\alpha$ relative to the *z* axis and is detected by a photodiode. If the optical axis of the linear polarizer is orthogonal to the electric field of the probe beam, i.e., when $\alpha = 90^{\circ }$, the transmitted signal results solely from the nonlinear FWM process.

The energy level diagram for the $F_g=3 \rightarrow F_e =2,3$, and 4 transitions of the ^85^Rb D2 line is shown in Fig. 1b. The probe and coupling beams are scanned across these transitions. The red arrows in Fig. 1b denote the transitions excited by laser beams, which are further discussed in the next subsection.

Figure 1c shows a part of the energy level diagram with Zeeman magnetic sublevels (Fig. 1b). To explain the mechanism underlying the generation of FWM signals, the electric field direction of the probe beam is selected as the quantization axis (i.e., the *z* axis). Then, the probe beam excites the transitions with $\Delta m=0$, whereas the coupling beam excites the transitions with $\Delta m =0$ and $\pm 1$. Here, $\Delta m$ is the difference in the magnetic quantum numbers between the excited and ground states. In Fig. 1c, the red and blue arrows denote excitations by the probe and coupling beams, respectively. The electric field of the probe beam after traversing the cell can be decomposed into two parts: one parallel ($E_{\Vert }$) and the other perpendicular ($E_{\bot }$) to the *z* axis, i.e., the polarization axis of the probe beam. Notably, $E_{\Vert }$ and $E_{\bot }$ are determined by the density matrix elements corresponding to $\Delta m =0$ and $\Delta m = \pm 1$, respectively. The probe beam cannot excite the $\Delta m = \pm 1$ transitions. Therefore, nonlinear processes are required to generate the $E_{\bot }$ component. An illustration of these phenomena is provided in Fig. 1c. The FWM process associated with one probe and two coupling photons generates $E_{\bot }$. To distinguish the $E_{\bot }$ component in the transmitted probe beam, the polarization transmission angle must be set to $\alpha = 90^{\circ }$.

### Calculation

We calculate the transmission of a probe beam, following its passage through an atomic cell and a linear polarizer, in the presence of a counter-propagating coupling beam for the $F_g=3 \rightarrow F_e =2,3$, and 4 transitions of the ^85^Rb D2 line. Notably, for the generation of FWM signals illustrated in Fig. 1c and d, the quantization axis is selected as the electric field direction of the probe beam. This choice is aimed at efficiently explaining the FWM signal generation mechanism. However, for computing the transmission and rotation angles of the probe beam, it is more convenient to select the propagation direction of the probe beam as the quantization axis. Thus, in this analysis, the polarization vector of the probe beam is expressed as $\hat{x} \cos \theta +\hat{y} \sin \theta = a_+ \hat{\varepsilon }_+ +a_0 \hat{\varepsilon }_0 +a_- \hat{\varepsilon }_-$ and that of the coupling beam is $\hat{x} = c_+ \hat{\varepsilon }_+ +c_0 \hat{\varepsilon }_0 +c_- \hat{\varepsilon }_-$, where $\hat{\varepsilon }_{\pm } = \mp 2^{-1/2} \left( \hat{x} \pm i \hat{y} \right)$ and $\hat{\varepsilon }_0 =\hat{z}$ are the spherical bases. The coefficients are defined as

2 $$\begin{aligned} a_{\pm 1}= & {} \mp 2^{-1/2} e^{\mp i \theta }, \quad a_0 =0, \nonumber \\ c_{\pm 1}= & {} \mp 2^{-1/2}, \quad c_0 =0. \end{aligned}$$

Owing to nonlinear interactions with atoms in the cell, the polarization of the probe beam is altered, i.e., the irradiance is reduced, and the polarization direction is rotated. When the susceptibility of the $\sigma ^\pm$ components of the probe beam is $\chi _{\pm }$, which is evaluated theoretically below considering all the magnetic sublevels, the transmission (*T*) and polarization rotation angle ($\eta$) can be expressed as^48^

3 $$\begin{aligned} T= & {} \frac{1}{2} \left( e^{-k \chi _+^i l} +e^{-k \chi _-^i l} \right) , \end{aligned}$$

4 $$\begin{aligned} \eta= & {} \frac{k}{4} \left( \chi _-^r -\chi _+^r \right) l, \end{aligned}$$

respectively, where $\chi _{\pm }^{r}$ ($\chi _{\pm }^{i}$) is the real (imaginary) part of $\chi _{\pm }$. In Eqs. (3) and (4), $k (= 2 \pi /\lambda )$ is the wave vector, $\lambda$ is the laser wavelength, and *l* is the length of the atomic cell. After traversing the cell, the polarization vector of the probe beam becomes

5 $$\begin{aligned} \hat{x} \cos \left( \theta + \eta \right) + \hat{y} \sin \left( \theta +\eta \right) , \end{aligned}$$

and the irradiance is reduced by a factor of *T*. Then, the probe beam passes through a polarizer with a polarization angle $\alpha$ relative to the *z*-axis. Thus, the detected signal at the photodiode is

6 $$\begin{aligned} T \times \cos ^2 \left( \eta - \alpha \right) . \end{aligned}$$

To determine *T* and $\eta$ in Eq. (6), it is necessary to find the susceptibilities $\chi _{\pm }$, which are composed of various density matrix elements. The density matrix elements can be obtained by solving the following density matrix equation in a frame rotating at the frequency of the coupling beam:

7 $$\begin{aligned} \dot{\rho }= -\frac{i}{\hbar } \left[ H_0 +V,\rho \right] +{\dot{\rho }}_\text{relax}, \end{aligned}$$

where $\rho$ is the density operator; and $H_0$ and *V* are the atomic and interaction Hamiltonians, respectively. In Eq. (7), $H_0$ and *V* can be written in the explicit form as

8 $$\begin{aligned} H_0= & {} -\sum _{m=-4}^4 \hbar \delta _2 \left| F_e =4, m \right\rangle \left\langle F_e =4, m \right| -\sum _{m=-3}^3 \hbar \left( \delta _2 +\Delta _{43} \right) \left| F_e =3, m \right\rangle \left\langle F_e =3, m \right| \nonumber \\{} & {} -\sum _{m=-2}^2 \hbar \left( \delta _2 +\Delta _{42} \right) \left| F_e =2, m \right\rangle \left\langle F_e =2, m \right| , \end{aligned}$$

9 $$\begin{aligned} V= & {} \frac{\hbar }{2} \sum _{q=-1}^{1} \sum _{F_e =2}^4 \sum _{m=-3}^3 (a_q \Omega _p e^{-i \delta _d t} +c_q \Omega _c) C_{3,m}^{F_e, m+q} \left| F_e,m+q \right\rangle \left\langle F_g, m \right| +\mathrm{h.c.}, \end{aligned}$$

In Eq. (8), $\delta _2 (=\delta +k v)$ is the effective detuning of the coupling beam experienced by an atom moving at velocity *v*, where $\delta$ represents the laser detuning. $\Delta _{4j}$ is the frequency spacing between states $\left| F_e =4 \right\rangle$ and $\left| F_e =j \right\rangle$, with $j=2$ and 3. In Eq. (9), $\Omega _p$ ($\Omega _c$) is the Rabi frequency of the probe (coupling) beam, and $C_{F_g, m_g}^{F_e, m_e}$ is the normalized transition strength between states $\left| F_e ,m_e \right\rangle$ and $\left| F_g ,m_g \right\rangle$^49^. $\delta _d (=-2 k v)$ is the effective detuning of the probe beam relative to that of the coupling beam, and h.c. denotes the harmonic conjugate. In Eq. (9), the coefficients $a_q$ and $c_q$ ($q=0,\pm 1$) are as defined in Eq. (2). The term ${\dot{\rho }}_\text{relax}$ in Eq. (7) represents the relaxation terms consisting of spontaneous emission and transit-time decay^50,51^.

Substitution of Eqs. (8) and (9) into Eq. (7) yields a set of coupled time-dependent differential equations. As discussed in our previous works^51,49^, to solve the differential equations in the steady-state regime, the density matrix elements should be decomposed into many Fourier components oscillating at specific frequencies. For example, when interactions of up to three photons are considered, the optical coherence, $\rho _{4,4;3,3} (\equiv \left\langle F_e =4, m_e=4 \right| \rho \left| F_g = 3, m_g =3 \right\rangle )$, can be decomposed as follows:

10 $$\begin{aligned} \rho _{4,4;3,3}= & {} \rho _{4,4;3,3}^{(1)} e^{-i \delta _d t}+\rho _{4,4;3,3}^{(2)}+\rho _{4,4;3,3}^{(3)} e^{i \delta _d t}+\rho _{4,4;3,3}^{(4)} e^{-2 i \delta _d t}. \end{aligned}$$

Other optical coherences, Zeeman coherences, and populations can be decomposed in an analogous method. Notably, $\rho _{F_e ,m \pm 1;3,m}^{(1)}$ with $F_e =2,3$, and 4 is responsible for the absorption and dispersion of the probe beam in Eq. (10). By introducing the decomposed density matrix elements into Eq. (7), along with $H_0$ and *V*, and selecting the terms oscillating at the same oscillation frequencies, a set of differential equations associated with expanded density matrix elements, such as $\rho _{4,4;3,3}^{(1)}$, is obtained. These equations are then solved in the steady-state regime to determine the Fourier components of the density matrix elements as functions of velocity and various detunings. Finally, the susceptibilities averaged over the Maxwell–Boltzmann velocity distribution are given by

11 $$\begin{aligned} \chi _{\pm } = -\frac{3\lambda ^3}{4 \pi ^2} \frac{N_\text{at} \Gamma }{a_{\pm 1}\Omega _1} \int _{-\infty }^{\infty } \frac{d v}{ \sqrt{\pi } v_\text{mp}}e^{-\left( v/v_\text{mp} \right) ^2} \sum _{F_e =2}^4 \sum _{m=-3}^3 C_{3,m}^{F_e, m \pm 1} \rho _{F_e,m \pm 1; 3,m}^{(1)}, \end{aligned}$$

where $N_\text{at}$ is the atomic number density in the cell, $\Gamma$ is the decay rate of the $5P_{3/2}$ state, and $v_\text{mp}$ denotes the most probable speed of the atoms. Then, the susceptibilities in Eq. (11) are used to determine *T* and $\eta$ in Eq. (6).

## Methods

**Figure 2:**
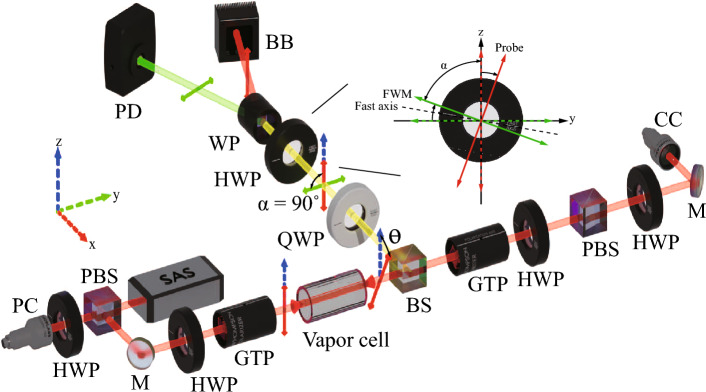
Schematic of the experimental setup. *HWP* half-wave plate, *QWP* quarter-wave plate, *BS* beam splitter, *PBS* polarizing beam splitter, *GTP* Glan–Thompson polarizer, *WP* Wollaston polarizer, *M* mirror, *BB* beam block, *PD* photodetector, *CC* coupling collimator, *PC* probe collimator.

The experimental setup is schematically illustrated in Fig. 2, involving a distributed Bragg reflector laser operating at 780 nm. The laser beam was coupled into single-mode polarization-maintaining fibers and divided into three paths using two polarizing beam splitters (PBSs). One path was used for the SAS setup, and the other two paths were used for the coupling and probe beams. The $1/e^2$ beam diameter of the Gaussian output beam from the fiber collimators was 5.7 mm, and the power of the probe and coupling beams was 1.0 mW. A ^85^Rb vapor cell with a length of 50 mm was placed at the center of three pairs of square Helmholtz coils ($570 \times 350 \times 265$ mm^3^) with 50 turns on each side to compensate for the Earth’s magnetic field. We have reduced the Earth’s magnetic field in the range of a few nT with a fluxgate magnetometer (Mag-01H, Bartington) because FWM signal generation is very sensitive to magnetic fields. The temperature of the vapor cell was $22\,^{\circ }\textrm{C}$. The probe and coupling beams were linearly polarized through a Glan–Thomson polarizer, and the beam power was controlled by a half-wave plate. The polarization direction of the probe beam was aligned with the *z*-axis. The angle $\theta$ between the linear polarization directions of the probe and coupling beams could be adjusted between 0° and 90°. At $\theta =0^{\circ }$ and $\theta =90^{\circ }$, the polarization of the coupling beam was horizontal and vertical to the probe polarization, respectively.

A Wollaston prism (WP) was used to spatially separate the vertical polarization of the probe beam and horizontal polarization of the generated FWM copropagating along the same beam path. Moreover, a quarter-wave plate was placed before the WP to compensate for elliptical polarization deviations arising from reflection on the beam splitters and transmission through the vapor cell. Unlike the previous analysis, in which the polarizer was rotated, here, a half-wave plate was used to rotate the linear polarization of the FWM signal to adjust the angle $\alpha$. This enabled the manipulation of the angle $\alpha$ between the vertical optical axis (*z*-axis) of the WP and linear polarization of the FWM signal. The measured extinction ratio of the FWM signal to the probe beam after passing through the WP was less than $<10^{-4}$.

## Results and discussion

**Figure 3:**
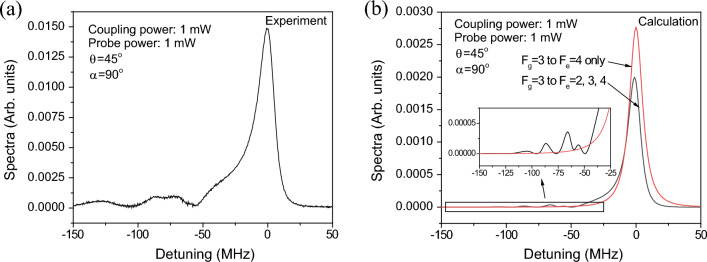
(**a**) Experimental and (**b**) calculated transmission spectra at $\theta =45^\circ$ and $\alpha =90^\circ$.

Figure 3 presents the experimentally obtained and calculated transmission spectra at $\theta =45^\circ$ and $\alpha =90^\circ$. The coupling and probe beams had power levels of 1 mW. In Fig. 3a, a broad and slightly asymmetric transmission signal can be observed around the $F_g=3 \rightarrow F_e=4$ resonance line, along with weak signals near a detuning of $-70$ MHz. The full-width-at-half-maximum (FWHM) of the FWM signal is approximately 16.9 MHz which is 2.78 times the natural linewidth of the transition. Because the power of 1 mW corresponds to the saturation parameter of 3.2 in the experiment, the probe and coupling beams may result in $\sim 2.7$ times the natural linewidth of the transition in the FWHM of the FWM signal. Thus, we find that the broadened FWM signal attributes to the power broadening due to the laser fields. Figure 3b shows the results calculated including all the $F_g=3 \rightarrow F_e=2,3$, and 4 transitions (black curve) and those calculated including only the $F_g=3 \rightarrow F_e=4$ transition line (red curve). The inset shows a detailed plot of the weak signals. The red curve exhibits a symmetric spectrum near the zero detuning, whereas the black curve displays an asymmetric spectrum. This asymmetry arises due to neighboring transitions, such as off-resonant FWM signals, resulting in a red-shift in the peak position and reduced peak height. The inset indicates that the weak signals near the detunings of $-60$ MHz and $-92$ MHz correspond to crossover signals for the $F_g=3 \rightarrow F_e=\{4,3\}$ and $F_g =3 \rightarrow F_e=\{4,2\}$ transitions, respectively. The smallness of the crossover signals results from the fact that the atoms moving at non-zero velocity contribute to the crossover signals.

**Figure 4:**
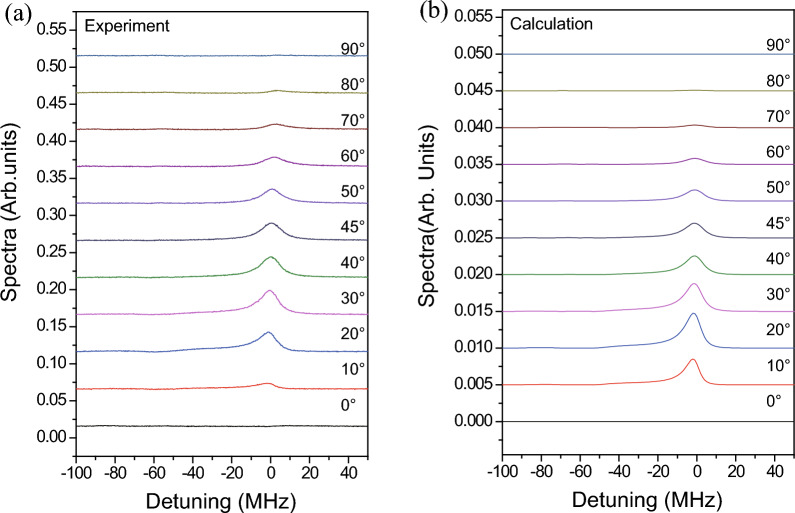
(**a**) Experimental and (**b**) calculated results for the dependence of the transmitted signals on angle $\theta$, with the laser power being 1 mW.

Figure 4 shows the dependence of the transmitted signals on the angle $\theta$, with $\alpha$ fixed at $90^{\circ }$. The experimental and calculated spectra are presented in Fig. 4a and b, respectively. The spectra for different angles of $\theta$ are shifted vertically to provide a clear view. The powers of the coupling and probe beams were 1.0 mW. As shown in Fig. 4, the experimental results are in excellent agreement with the calculated results. As $\theta$ increases, the transmitted signal increases up to $\theta =30^{\circ }$ and then monotonically decreases. The FWM signals vanish when $\theta =0^{\circ }$ and $90^{\circ }$. When $\theta =0^{\circ }$, both the probe and coupling photons excite only the $\Delta m= 0$ transitions. Thus, the density matrix elements satisfying $\Delta m= \pm 1$ do not exist, resulting in the absence of the FWM signals. In addition, when $\theta =90^{\circ }$, the probe and coupling photons excite the $\Delta m= 0$ and $\Delta m= \pm 1$ transitions, respectively. Because excitations of $\Delta m= 0$ by the coupling photon are necessary to generate the FWM signals, as shown in Fig. 1d, the FWM signals cannot be observed when $\theta =90^{\circ }$.

**Figure 5:**
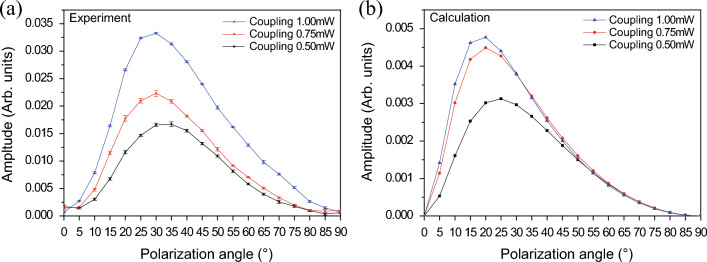
(**a**) Experimental and (**b**) calculated results of the maximum amplitude of the transmitted signal depending on the angle $\theta$. The probe beam power is fixed at 1.0 mW, and the coupling beam power levels are 0.5, 0.75, and 1.0 mW.

Figure 5 shows the maximum amplitude of the transmitted signal at various $\theta$ values. The experimental and calculated results are shown in Fig. 5a and b, respectively. In Fig. 5, the results at coupling-beam power levels of 0.5, 0.75, and 1.0 mW are displayed as black, blue, and red dots, respectively, with the probe beam power fixed at 1.0 mW. As expected, the signal amplitude increases as the coupling-beam power ($P_\text{c}$) increases. As shown in Fig. 5a, the angles $\theta$ corresponding to the maximum amplitude at $P_\text{c}=0.5$, 0.75, and 1.0 mW are approximately $35^\circ$, $30^\circ$, and $30^\circ$, respectively. As $P_\text{c}$ increases, the maximum angle slightly decreases. This trend is consistently reproduced in the calculated results, as shown in Fig. 5b. Although the experimental results are in agreement with the theoretical results, some minor discrepancies persist. In the experiment, the laser beam intensity profile was assumed to be Gaussian, whereas it was considered to be constant in theory. Therefore, the discrepancies between the experimental and theoretical results may be attributed to this difference.

We observed that the angle $\theta$ corresponding to the maximum transmitted signal decreases as the laser beam power increases. In addition, we found separately this angle increases and approaches $45^\circ$ as the laser beam power becomes reduced. This phenomena can be understood by simply considering the routes of the interactions of the components of the coupling beam shown in the diagram in Fig. 1d, and the fact that the Rabi frequencies of the $\pi$ and $\sigma ^{\pm }$ components are proportional to $\cos \theta$ and $\sin \theta$, respectively. When the coupling power is very weak, there are two routes of the components of the coupling beam responsible for the generation of FWM signal. Thus, the signal is proportional to the square of $2 \Omega _c^2 \cos \theta \sin \theta$, which is maximized at $\theta = 45^\circ$. As the coupling beam power increase, more routes of interactions of the components are involved in the generation of the signal. When we consider the interaction of the order of $\Omega _c^{2m}$ with *m* the integer, which implies the 2*m* components of the coupling beam are involved in the interaction, it is easy to find that the signal is proportional to the square of the following:

12 $$\begin{aligned} \sum _{j=0}^{m-1} 2 \left( m - j \right) \left( \cos \theta \right) ^{2m-1-2j} \left( \sin \theta \right) ^{2 j+1} \, \Omega _c^{2m}. \end{aligned}$$

For example, the FWM signal at $m=2$ is proportional to the square of $4 \cos \theta ^3 \sin \theta +2 \cos \theta \sin ^3 \theta$, which is maximized at $\theta = 36.8^\circ$. It can be readily seen that the angle $\theta$ for the maximum transmitted signal decreases as *m* increases. Thus, we can conclude that as the coupling beam power increases, the $\pi$ component of the coupling beam contribute more significantly than the $\sigma ^{\pm }$ component, and accordingly the angle $\theta$ for the maximum transmitted signal decreases.

**Figure 6:**
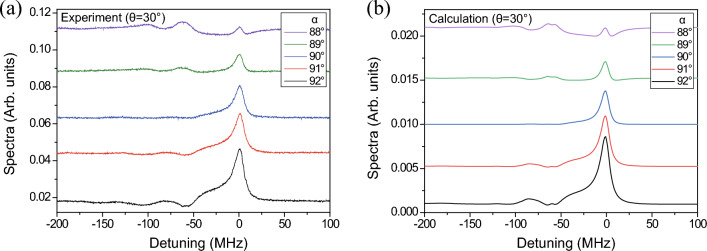
(**a**) Experimental and (**b**) calculated results of the dependence of the transmission signals on the angle $\alpha$.

Thus far, we measured the transmitted signals perpendicular to the electric field of the probe beam, i.e., with $\alpha =90^{\circ }$. In this configuration, only the pure FWM signal is detected. Next, we examine the variation in the signal when the probe beam passes through a linear polarizer with a polarization angle different from $90^\circ$. The experimental and calculated results at $\theta =30^{\circ }$ are shown in Fig. 6. The results at angles $\alpha =88^{\circ }$, $89^{\circ }$, $90^{\circ }$, $91^{\circ }$, and $92^{\circ }$ are displayed from top to bottom. As in Fig. 6, the spectra for different angles of $\alpha$ are shifted vertically to provide a clear view. The experimental and calculated results demonstrated excellent agreement. The transmission spectra at $\alpha \ne 90^{\circ }$ include the signals resulting from the density matrix elements with $\Delta m =0$ as well as FWM signals.

The resonance signals at zero detuning exhibit significant variations for different $\alpha$ values. The signals become broader for $\alpha > 90^{\circ }$ and weaker for $\alpha < 90^{\circ }$. The crossover signal for the $F_g =3 \rightarrow F_e =\{4,3 \}$ transition is observed near the detuning of $-60$ MHz, but that for the $F_g =3 \rightarrow F_e =\{4,2 \}$ transition spans a broad range of detunings from $-80$ to $-100$ MHz. The reason for this variation in the detuning of the crossover signal for the $F_g =3 \rightarrow F_e =\{4,2 \}$ transition is not entirely clear. However, we hypothesize that the complex polarization may contribute to this variation. Moreover, the crossover signals are significant when $\alpha \ne 90^{\circ }$. This observation can be explained in terms of the average transition strengths ($S_0$ and $S_{\pm 1}$) for signals generated from the density matrix elements satisfying $\Delta m=0$ and $\Delta m=\pm 1$, respectively:

$$\begin{aligned} S_0= & {} \sum _{m=-3}^{3} \left( C_{3,m}^{4,m} \left( C_{3,m}^{3,m}\right) ^2 \right) ^2 +\left( C_{3,m}^{3,m} \left( C_{3,m}^{4,m}\right) ^2 \right) ^2, \\ S_{\pm 1}= & {} \sum _{m=-3}^{3} \left( C_{3,m}^{4,m} C_{3,m}^{3,m+1} C_{3,m+1}^{3,m+1} \right) ^2 +\left( C_{3,m}^{3,m} C_{3,m}^{4,m+1} C_{3,m+1}^{4,m+1} \right) ^2. \end{aligned}$$

According to these expressions, $S_0 / S_{\pm 1} \simeq 2.0$. The crossover signals at $\alpha = 90^{\circ }$ consist of only the term associated with $S_{\pm 1}$, whereas those at $\alpha \ne 90^{\circ }$ consist of both the terms associated with $S_{\pm 1}$ and $S_{0}$. Thus, the crossover signals are enhanced when $\alpha \ne 90^{\circ }$.

In conclusion, we explore the generation of polarization-selective FWM signals in a conventional coupling-probe spectroscopy setup for $^{85}$Rb atoms. This setup includes two counter-propagating laser beams in a degenerate multi-level atomic system, corresponding to the $F_g=3 \rightarrow F_e =2,3,4$ transitions of the $^{85}$Rb D2 line. We selectively obtain the pure FWM signals in the transmitted probe beam, which pertain to the component perpendicular to the polarization direction of the input probe beam. In addition, we demonstrate the asymmetric spectrum of the FWM signal, which can be attributed to the neighboring transition effect. The experimental results exhibit excellent agreements with the theoretical results.

To this point, our analysis focuses on the degenerate Zeeman magnetic sublevels to explain the generation of FWM signals in coupling-probe spectroscopy. As is well-known, FWM signals must satisfy phase-matching conditions, such as the polarization, frequency, and propagating direction, and are thus sensitive to external magnetic fields that induce shifts in the Zeeman magnetic sublevels. Therefore, our system could be applied for magnetic sensing in near-zero-field conditions, such as in atomic magnetometers based on coherent population trapping ^52^, which are simple yet robust. The experimental work on magnetic sensing is currently under progress. Recently, Lee et al.^53,54^ demonstrated high-performance laser frequency stabilization through modulation transfer spectroscopy (MTS). MTS can serve as a self-complete frequency standard for mobile ultra-sensitive absolute atom interferometers. Some studies theoretically demonstrated the effect of coherent population trapping on MTS signals^55,56^. Although researchers have focused on the beat signal of probe beams and FWM signals, direct observation of the FWM signals within the MTS signal has not been conducted. Our research provides opportunities for investigating the direct characteristics of FWM signals in MTS frameworks, thereby enhancing our understanding of the reported results. Furthermore, this study offers valuable insights into various Doppler-free spectroscopic techniques that have been previously explored.

## Data Availability

The datasets used and/or analysed during the current study available from the corresponding author on reasonable request.
